# Acellular Extracellular Matrix Bioscaffolds for Cardiac Repair and Regeneration

**DOI:** 10.3389/fcell.2019.00063

**Published:** 2019-04-26

**Authors:** Simranjit S. Pattar, Ali Fatehi Hassanabad, Paul W. M. Fedak

**Affiliations:** Section of Cardiac Surgery, Department of Cardiac Science, Cumming School of Medicine, Libin Cardiovascular Institute of Alberta, University of Calgary, Calgary, AB, Canada

**Keywords:** extracellular matrix, myocardial infarction, heart failure, cardiac surgery, bio-scaffold, biomaterials, surgical revascularization, cardiac fibroblast

## Abstract

Heart failure is a progressive deterioration of cardiac pump function over time and is often a manifestation of ischemic injury caused by myocardial infarction (MI). Post-MI, structural remodeling of the infarcted myocardium ensues. Dysregulation of extracellular matrix (ECM) homeostasis is a hallmark of structural cardiac remodeling and is largely driven by cardiac fibroblast activation. While initially adaptive, structural cardiac remodeling leads to irreversible heart failure due to the progressive loss of cardiac function. Loss of pump function is associated with myocardial fibrosis, wall thinning, and left ventricular (LV) dilatation. Surgical revascularization of the damaged myocardium via coronary artery bypass graft (CABG) surgery and/or percutaneous coronary intervention (PCI) can enhance myocardial perfusion and is beneficial. However, these interventions alone are unable to prevent progressive fibrotic remodeling and loss of heart function that leads to clinical end-stage heart failure. Acellular biologic ECM scaffolds can be surgically implanted onto injured myocardial regions during open-heart surgery as an adjunct therapy to surgical revascularization. This presents a novel therapeutic approach to alter maladaptive remodeling and promote functional recovery. Acellular ECM bioscaffolds have been shown to provide passive structural support to the damaged myocardium and also to act as a dynamic bioactive reservoir capable of promoting endogenous mechanisms of tissue repair, such as vasculogenesis. The composition and structure of xenogenic acellular ECM bioscaffolds are determined by the physiological requirements of the tissue from which they are derived. The capacity of different tissue-derived acellular bioscaffolds to attenuate cardiac remodeling and restore ECM homeostasis after injury may depend on such properties. Accordingly, the search and discovery of an optimal ECM bioscaffold for use in cardiac repair is warranted and may be facilitated by comparing bioscaffolds. This review will provide a summary of the acellular ECM bioscaffolds currently available for use in cardiac surgery with a focus on how they attenuate cardiac remodeling by providing the necessary environmental cues to promote endogenous mechanisms of tissue repair.

## Introduction to Heart Failure

According to the American Heart Association (AHA), the prevalence of congestive heart failure (CHF) is expected to increase by 46% from 2012 to 2030 with a staggering 960,000 new CHF cases reported annually (Benjamin et al., 2017). CHF is a progressive condition in which cardiac pump function deteriorates over time. Heart failure is often a manifestation of an ischemic injury to the heart caused by myocardial infarction (MI) (Velagaleti et al., 2008). Following an MI, structural remodeling of the infarcted myocardium ensues (Sutton and Sharpe, 2000; Fedak et al., 2005a,b). Initially, structural cardiac remodeling is adaptive, with the aim of preventing ventricular free wall rupture. However, prolonged structural cardiac remodeling leads to irreversible heart failure due to the progressive loss of cardiac function (Fedak et al., 2005a,b). Heart function deterioration results from myocardial fibrosis, wall thinning, and left ventricular (LV) dilatation (Fedak et al., 2005a,b; Fraccarollo et al., 2012; Xie et al., 2013).

## Heart Failure Management and Bioscaffolds

There are a plethora of pharmacological interventions, including antianginal medications such as beta blockers, which may be employed to improve the symptoms and prognosis of heart failure patients. However, pharmacotherapy alone is unable to elicit functional recovery of the infarcted myocardium (McDonald et al., 2017; Patel et al., 2017). Revascularization of the infarcted myocardium is currently achieved either through coronary artery bypass graft (CABG) surgery and/or percutaneous coronary intervention (PCI). While these approaches are indicated for improving morbidity and mortality, they are unable to prevent progressive cardiac fibrosis and structural remodeling which leads to end-stage heart failure (McDonald et al., 2017). CREDO-Kyoto (Coronary REvascularization Demonstrating Outcome Study in Kyoto) is a three-year outcome study of the PCI/CABG Registry Cohort. It revealed that of 392 patients undergoing CABG 12% were readmitted for heart failure and 4% required repeat revascularization. Similarly, of 672 patients undergoing PCI 22% were readmitted for heart failure and 19% required repeat revascularization (Marui et al., 2014).

In the past, the field of cardiovascular tissue engineering has focused on leveraging a stem cell-based or gene therapy-based approach as a solution to restoring cardiac function post-MI. However, the road to clinical translation of these approaches has largely been fraught with challenges, specifically uncertainty regarding clinical efficacy and feasibility (Balsam et al., 2004; Murry et al., 2004; Gyöngyösi et al., 2015; Kaiser, 2018; Reardon, 2018). Acellular extracellular matrix (ECM) bioscaffolds are a new and innovative solution being investigated as an adjunct therapy to the surgical revascularization of damaged myocardium (Mewhort et al., 2014, 2016b, 2017; Park et al., 2017; Svystonyuk et al., 2018). These acellular ECM bioscaffolds may hold the key to unlocking the potential of endogenous mechanisms of tissue repair and regeneration at the site of an MI. By providing the necessary environmental cues, the ultimate aim of acellular ECM bioscaffold therapy is to promote functional recovery of the damaged myocardium. In this review, we provide a summary of the acellular ECM bioscaffolds currently used and in development for use in cardiac surgery. Better understanding of this promising therapeutic strategy may facilitate its transition into clinical practice.

## The Central Role of the Cardiac Fibroblast

**FIGURE 1:**
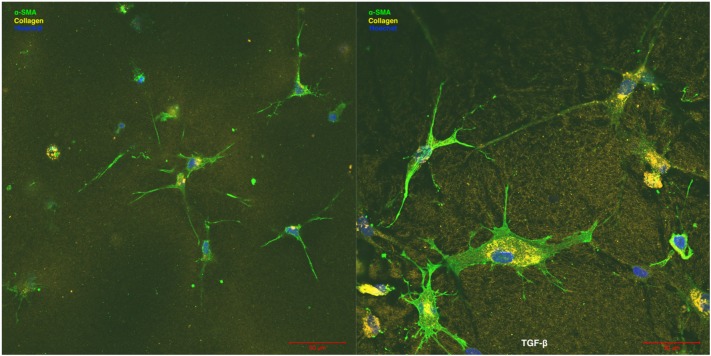
Human cardiac fibroblast (left) compared to TGF-β activated human cardiac myofibroblasts (right). Myofibroblasts are larger in cell size, have an increased number of cell extensions, and increased cell extension length. Alpha smooth muscle actin (α-SMA) expression and collagen production and deposition (collagen, yellow) are both increased in human cardiac myofibroblasts compared to human cardiac fibroblasts. Hoechst staining is used to visualize cell nuclei (nuclei, blue). Images were provided courtesy of Dr. Guoqi Teng, University of Calgary.

Our research group is particularly interested in the central role that the cardiac fibroblast plays in maladaptive structural cardiac remodeling and the progression of cardiac fibrosis (Mewhort et al., 2014, 2016a,b, 2017; Svystonyuk et al., 2015, 2018; Teng et al., 2015; Park et al., 2017). As one of the predominant non-cardiomyocyte cell types that comprise the heart, cardiac fibroblasts contribute to the structural, mechanical, biochemical, and electrical properties of the heart (Camelliti et al., 2005; Souders et al., 2009). By responding to changes in the microenvironment, cardiac fibroblasts play a key role in maintaining ECM homeostasis via directly altering the synthesis and degradation of the cardiac ECM components (Fedak et al., 2005a,b; Krenning et al., 2010; Fan et al., 2012). The maintenance and regulation of the ECM is essential as it forms a structural network that supports and interconnects the cardiac cells. Further, the biochemical role of cardiac fibroblasts further impacts cardiac function as they secrete a diverse array of growth factors and ECM-regulatory proteins (Fedak et al., 2005a,b). For instance, angiogenesis is in part driven by a paracrine interaction amongst cardiac fibroblasts and neighboring endothelial cells which is mediated through fibroblast growth factor-2 (FGF-2) and vascular endothelial growth factor (VEGF) (Zhao and Eghbali-Webb, 2001). Finally, cardiac fibroblasts also play a role in establishing the normal electrophysiology of the heart. The electrical coupling of cardiac fibroblasts to cardiomyocytes via gap junction channel proteins connexin-43 and connexin-45 has been characterized *in-vitro* (Camelliti et al., 2004; Mahoney et al., 2016). In the event of ischemic injury and the resulting disruption of the local microenvironment of the infarcted myocardium, cardiac fibroblasts become activated to a myofibroblast state (Baum and Duffy, 2011; Dixon and Wigle, 2015; Figure 1). Myofibroblast activity drives maladaptive structural cardiac remodeling and fibrosis through dysregulation of ECM homeostasis and disruption of the local bioactive milieu, including growth factors and cytokines (Fedak et al., 2005a,b; Krenning et al., 2010; Dixon and Wigle, 2015).

## Myofibroblast-Mediated Extracellular Matrix Remodeling

**FIGURE 2:**
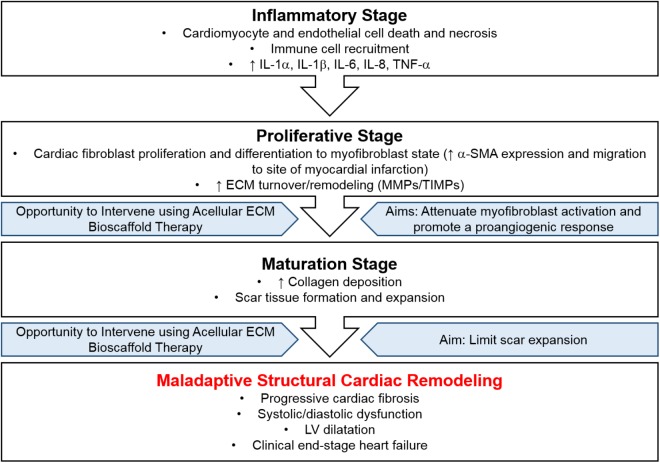
Disease progression following myocardial infarction. The infarcted myocardium undergoes a three-stage wound healing process, (1) inflammatory stage, (2) proliferative stage, and (3) maturation stage, resulting in maladaptive structural cardiac remodeling. Intervention using acellular ECM bioscaffold therapy may be used to attenuate myofibroblast activation, promote a proangiogenic response, and limit scar expansion.

Specifically, the infarcted myocardium undergoes a three-stage wound healing process: (1) inflammatory stage, (2) proliferative stage, and (3) maturation stage (Figure 2). Initially, the inflammatory stage is characterized by cardiomyocyte and endothelial cell death, immune cell recruitment, and an increase in pro-inflammatory cytokines (Dobaczewski et al., 2010b; Shinde and Frangogiannis, 2014; Saxena and Frangogiannis, 2015). During this stage, cardiac fibroblasts assume a pro-inflammatory phenotype and contribute to inflammation via the production of various cytokines (IL-1α, IL-1β, IL-6, IL-8, and TNF-α) (Kawaguchi et al., 2011; Fan et al., 2012; Shinde and Frangogiannis, 2014). Next, the proliferative stage is marked by cardiac fibroblast differentiation to a myofibroblast phenotype and migration to the region of infarcted myocardium (Shinde and Frangogiannis, 2014; Figure 2). This shift may be driven by an upregulation in transforming growth factor beta (TGF-β), ED-A fibronectin, and mechanical stress (Serini et al., 1998; Lee et al., 1999; Vaughan et al., 2000; Tomasek et al., 2002; Zhao et al., 2007; Dobaczewski et al., 2010a; Shinde and Frangogiannis, 2014; Figure 1). Myofibroblasts are characterized by increased alpha-smooth muscle actin (α-SMA) expression (Figure 1), which corresponds with increased contractility and manipulation of the surrounding ECM environment (Leslie et al., 1991; Arora and McCulloch, 1994; Hinz et al., 2001). Myofibroblasts also display altered matrix metalloproteinase (MMP) and tissue inhibitors of matrix metalloproteinases (TIMPs) production (Fedak et al., 2005b). The altered expression of these ECM-regulatory proteins results in the net deposition of type I collagen, along with other ECM proteins (Brown et al., 2005; Heymans et al., 2005). Finally, the purpose of the maturation stage is scar tissue formation, wherein increased ECM deposition is necessary to form a collagenous scar and to prevent ventricular free wall rupture at the site of MI (Camelliti et al., 2005; Souders et al., 2009; Figure 2). It is in this reparative manner that myofibroblast activity and structural cardiac remodeling is initially adaptive.

However, persistent myofibroblast activation eventually leads to maladaptive scar thickening and expansion the result of which is mechanical stiffness, systolic and diastolic dysfunction, and progressive cardiac fibrosis (Figure 2). Regional and temporal changes in myocardial contractility following ischemic injury to the heart allow the local region of MI to be differentiated from the surrounding border zone and the remote unaffected myocardial tissue (Jackson et al., 2002; Park et al., 2006; Shimkunas et al., 2013; Arunachalam et al., 2018; Torres et al., 2018). In particular, the border zone is a normally perfused region of hypocontractility, where contractility has been shown to be reduced by 50% compared to that of the remoted unaffected zone *in-vivo*, and plays a central role in infarct expansion (Jackson et al., 2002; Blom et al., 2007; Shimkunas et al., 2011; Richardson and Holmes, 2015). Overall, the systolic and diastolic dysfunction that arises due to disrupted myocardial contractility contributes to poor cardiac pump function and may lead to clinical end-stage heart failure. Characterization of cardiac tissue obtained from patients undergoing heart transplantation as a result of clinical end-stage heart failure reveals a 36% reduction in the number of LV cardiomyocytes and a concomitant 28% increase in collagen content within this region (Beltrami et al., 1994). Furthermore, LV chamber volume is increased 4.6-fold, in conjunction with a 56% reduction in LV mass-to-chamber volume ratio (Beltrami et al., 1994).

While activation of the cardiac fibroblast to a myofibroblast state is central to the dysregulation of the cardiac ECM and the structural cardiac remodeling that ensues, it is important to note that these cells do not function in isolation. Cardiomyocytes, endothelial cells, and immune cells also play an important role in determining the structural, mechanical, biochemical, and electrical properties of the heart. However, as sentinel cells of the cardiac ECM, cardiac fibroblasts respond to a plethora of stimuli and modulate the function of the surrounding cardiac cells (Baudino et al., 2006; Souders et al., 2009; Saxena and Frangogiannis, 2015). Therefore, therapeutic solutions aimed at attenuating maladaptive structural cardiac remodeling should first focus on myofibroblast-mediated ECM remodeling.

## Acellular Extracellular Matrix Bioscaffolds as a Therapeutic Solution

The ability to restore an optimal ECM microenvironment presents a therapeutic opportunity by which maladaptive structural cardiac remodeling may be influenced. Acellular ECM bioscaffolds can be leveraged to provide the necessary environmental cues to attenuate cardiac fibroblast activation, thereby preventing excessive infarct-derived scar expansion and thickening. These bioscaffolds may also promote endogenous mechanisms of repair and regeneration, such as angiogenesis or vasculogenesis, by way of their bioinductive properties (Mewhort et al., 2014, 2016b, 2017; Park et al., 2017; Svystonyuk et al., 2018). While an array of synthetic scaffold materials are also in development, this review will focus on acellular biologic ECM scaffolds used or in development for use in cardiac surgery post ischemic injury. The composition and structure of acellular ECM bioscaffolds are a function of the physiological requirements of the tissue from which they are derived. As the healthy cardiac ECM microenvironment is a complex milieu of ECM components, cardiac cells, growth factors, cytokines, and matricellular proteins, it stands to reason that a biologic ECM scaffold may provide additional benefits as it best approximates this complex and diverse ECM microenvironment.

Acellular ECM bioscaffolds may be differentiated by, (1) the tissue source from which they are derived, and (2) the fixation method, or lack thereof, implemented in processing the ECM bioscaffold (Table 1). Acellular porcine-derived small intestine submucosa (SIS) ECM and acellular bovine-derived pericardium (BP) ECM bioscaffolds have been the focal point of clinical investigations for use in cardiac surgery. Notably, while proprietary techniques of glutaraldehyde fixation are most commonly utilized for the fixation or cross-linking of most bioscaffolds, CorMatrix^®^ is non-crosslinked (non-fixed) and PhotoFix^TM^ is cross-linked using dye-mediated photo-oxidation (Table 1). Further, while the methods and intricacies regarding the decellularization of these bioscaffolds are beyond the scope of this review, many patented techniques exist to obtain an acellular ECM bioscaffold. The goal of decellularization is to achieve an acellular tissue-derived ECM bioscaffold from which the antigenic epitopes associated with cell membranes and intracellular components have been removed; this minimizes the possibility of an adverse immunogenic response by recipients of the bioscaffold (Badylak et al., 2015).

**Table 1:** Partial list of commercially available ECM bioscaffolds for potential use in cardiac repair and regeneration.

Bioscaffold Product	Company	Donor Source	Tissue Source	Fixation Method
AltiPly^®^	Aziyo Biologics, Inc.	Human	Placental	Natural
CardioCel	Admedus Ltd.	Bovine	Pericardium	ADAPT^®^, glutaraldehyde-based
CorMatrix^®^	CorMatrix Cardiovascular, Inc.	Porcine	Small intestinal submucosa (SIS)	Natural
Edwards Pericardial Patch	Edwards Lifesciences Corp.	Bovine	Pericardium	XenoLogiX^TM^, glutaraldehyde-based
Matrix Patch^TM^	Auto Tissue Berlin GmbH	Equine	Pericardium	–
Miroderm^®^	Miromatrix Medical, Inc.	Porcine	Liver	Natural
No-React^®^ Pericardial Patch	BioIntegral Surgical, Inc.	Bovine	Pericardium	No-React^®^, glutaraldehyde-based
NuShield^®^	Organogenesis, Inc.	Human	Placenta	–
Peri-Guard^®^	Synovis Life Technologies, Inc.	Bovine	Pericardium	Glutaraldehyde-based
PhotoFix^TM^	Cryolife, Inc.	Bovine	Pericardium	Dye-mediated photo-oxidation
SJM^TM^ Pericardial Patch	St. Jude Medical, Inc.	Bovine	Pericardium	EnCap^TM^, glutaraldehyde-based
Tyke^®^	Aziyo Biologics, Inc.	Porcine	Small intestinal submucosa (SIS)	Natural
Vascutek Pericardial Patch	Vascutek Ltd.	Porcine	Pericardium	Glutaraldehyde-based
XenoSure^®^	LeMaitre^®^ Vascular	Bovine	Pericardium	Glutaraldehyde-based


*–, information not available.*

## Acellular Small Intestine Submucosa Derived Bioscaffolds

**FIGURE 3:**
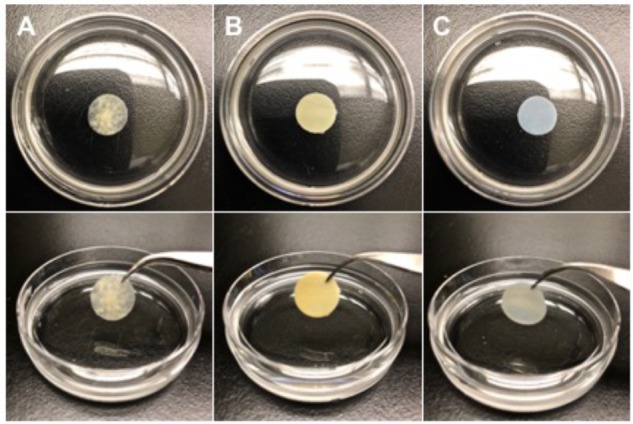
Representative images of three acellular extracellular matrix bioscaffolds (1.2 × 1.2 cm cut-outs). **(A)** CorMatrix^®^ (CorMatrix Cardiovascular, Inc., United States) porcine small intestine submucosa (SIS), natural, rehydrated in saline solution **(B)** Peri-Guard^®^ (Synovis Life Technologies, Inc., United States) bovine pericardium, glutaraldehyde cross-linked, and **(C)** PhotoFix^TM^ (Cryolife, Inc., United States) bovine pericardium, dye-mediated photo-oxidation cross-linked.

Our research group has extensively characterized the therapeutic benefit of CorMatrix^®^ - SIS-ECM (CorMatrix Cardiovascular, Inc., United States) for cardiac repair (Figure 3A). As the small intestine is a highly vascularized organ, it is unsurprising that SIS-ECM has been shown to contain essential proangiogenic growth factors, including FGF-2, VEGF, and HGF (Badylak, 2002; Mewhort et al., 2017). *In-vitro* characterization using human cardiac fibroblasts has demonstrated that CorMatrix^®^ promotes an enhanced FGF-2 dependent cell-mediated proangiogenic response (Mewhort et al., 2017). Further, CorMatrix^®^ enhances new blood vessel assembly both *in-vitro* using human umbilical vein endothelial cells (HUVEC) and *in-vivo* using a rat MI model (Mewhort et al., 2017). Using both a rat MI model and a pre-clinical porcine model of coronary ischemia-reperfusion, our research group has also shown that infarct repair using CorMatrix^®^ attenuates myocardial infarct expansion and elicits functional recovery (Mewhort et al., 2016b, 2017). Specifically, surgical implantation of CorMatrix^®^ sized to the area of infarcted myocardium displayed an improvement in ejection fraction and a trend toward improved LV compliance compared to sham, as measured by end-diastolic pressure-volume relationship (EDPVR) (Mewhort et al., 2017).

A recent systematic review of the initial preclinical and clinical findings of CorMatrix^®^ highlights the therapeutic potential of CorMatrix^®^, but also underscores the need for further elucidation of its specific surgical indications (Mosala Nezhad et al., 2016). For instance, a prospective multicenter clinical study, including 103 patients that received CorMatrix^®^ implants (38 – valve repair; 16 – septal reconstruction; 71 – arterial plasty; and 7 – other use), found no detectable calcification of the bioscaffold in patients at reoperation, follow-up imaging, or medium-term histological analysis after explanation (Padalino et al., 2015). As calcification is a primary drawback of glutaraldehyde fixed acellular ECM bioscaffolds, CorMatrix^®^ shows significant promise in overcoming this translational hurdle. However, CorMatrix^®^ has been shown to elicit an inflammatory response particularly in pediatric populations undergoing mitral or aortic valvuloplasties, which may be driven by the presence of the porcine α-gal epitope (Zaidi et al., 2014; Rosario-Quinones et al., 2015). It also remains unclear whether or not the bioscaffold itself is capable of remodeling and integrating into the native cardiac environment (Zaidi et al., 2014; Padalino et al., 2015). Extracardiac applications or applications in low-mechanical force cardiac environments may be the most suitable surgical indication for CorMatrix^®^ (Mosala Nezhad et al., 2016). There is value in further exploring the clinical potential of CorMatrix^®^, where more histopathological assessment of explanted bioscaffolds could be particularly beneficial. Our group is presently completing the first-in-man clinical feasibility pilot trial (NCT02887768) where acellular ECM bioscaffold therapy, CorMatrix^®^, is surgically implanted at the time of CABG surgery.

## Acellular Pericardium Derived Bioscaffolds

Bovine-derived pericardium (BP) ECM bioscaffolds have a wide variety of uses in cardiac surgery, including but not limited to pericardial closure, valve repair and replacement, and septal reconstruction. Glutaraldehyde cross-linked BP-ECM bioscaffolds, such as Peri-Guard^®^ (Synovis Life Technologies Inc., United States; Figure 3B) offer great utility in cardiac surgery due to their predictable and robust nature (Schmidt and Baier, 2000; Dunn, 2012). However, there are significant challenges associated with glutaraldehyde fixation, including calcification, the presence of an inflammatory reaction, and the cytotoxic nature of glutaraldehyde itself (Golomb et al., 1987; Dunn, 2012; van Steenberghe et al., 2017).

A variety of alternative strategies have emerged to neutralize or remove glutaraldehyde from cross-linked BP-ECM bioscaffolds, or to replace the use of glutaraldehyde entirely. PhotoFix^TM^ (Cryolife Inc., United States; Figure 3C), is a BP-ECM bioscaffold that is cross-linked using a proprietary dye-mediated photo-oxidation technique as an alternative to glutaraldehyde cross-linking. PhotoFix^TM^ has been shown to be non-immunogenic and non-calcific when compared to glutaraldehyde cross-linked counterparts *in-vivo*, using rat, rabbit, and sheep models (Bianco et al., 1996; Moore and Phillips, 1997; Moore et al., 1998). In addition, PhotoFix^TM^ demonstrates an ability to integrate into the native cardiac environment by supporting cell adhesion and migration, as evidenced by partial endothelialization two-years post implantation (Bianco et al., 1996; Moore and Phillips, 1997; Moore et al., 1998; Schmidt and Baier, 2000). Recently, a retrospective study including 364 patients, median age 5.3 years (range, < 1 months to 56 years), who received PhotoFix^TM^ implants (insertion site, 149 – right ventricular outflow tract; 168 – pulmonary artery; 21 – valve repair; 81 – septal reconstruction; 16 – superior vena cava reconstruction; 26 – aortic arch; and 29 – other), found that at follow-up 3.2 ± 1.6 years post-implantation there were no deaths related to PhotoFix^TM^ bioscaffold failure (Baird et al., 2016). Moreover, histopathology of explanted PhotoFix^TM^ bioscaffolds revealed in many cases absent or minimal inflammation and calcification (Baird et al., 2016). Currently, the clinical benefits of PhotoFix^TM^ in vascular repair or reconstruction surgery are being explored in the ongoing post-market, prospective evaluation of PHOTO-oxidized Bovine Pericardium in Vascular Surgery (PHOTO-V) clinical trial (NCT03669042). While this study is powered to assess outcomes in patients undergoing vascular repair or reconstruction surgery, it may provide additional insight into the use of PhotoFix^TM^ as an adjunct therapy to the surgical revascularization of infarcted myocardium post MI.

CardioCel^®^ (Admedus Limited, Australia), is a BP-ECM bioscaffold that is cross-linked using a proprietary fixation technique known as ADAPT^®^ (Pavy et al., 2017). Although this technique utilizes glutaraldehyde fixation, it employs a low concentration of glutaraldehyde (0.05%) compared to other clinical standards such as Peri-Guard (0.6%) (Neethling et al., 2018). Additionally, CardioCel^®^ ADAPT^®^ involves an additional detoxification step to remove any traces of glutaraldehyde (Pavy et al., 2017). Recently, *in-vivo* characterization of calcification compared CardioCel^®^ to Peri-Guard^®^, PhotoFix^TM^, and CorMatrix^®^, using a juvenile subcutaneous rat model. While a significant difference in calcification was not seen across groups, the extractable calcium level of CardioCel^®^ was similar to that of PhotoFix^TM^, 0.45 and 0.44 μg/mg tissue, respectively, and was lower than Peri-Guard^®^, 0.85 μg/mg tissue (Neethling et al., 2018). Variable outcomes have been shown *in-vivo;* CardioCel^®^ displayed promising endothelialization and remodeling in the native cardiac ECM environment and resistance to calcification in a chronic juvenile sheep model of pulmonary valve and mitral valve reconstruction (Brizard et al., 2014). However, when used as an implant in the ascending aorta and/or pulmonary artery in a juvenile porcine model CardioCel^®^ displayed poor integration or remodeling in the native ECM cardiac environment and significant calcification (Salameh et al., 2018). Similar trends have been observed in human pediatric patients where histopathological analysis of explanted CardioCel^®^ reveals early graft failure due to calcification and intimal thickening in high pressure areas (aortic arch position) (Pavy et al., 2017). Notably, CardioCel^®^ was well tolerated in lower pressure areas (septal, valvar, and pulmonary artery positions) (Pavy et al., 2017). Given its potential, these results clearly highlight the need for continued investigation of the optimal surgical indications of CardioCel^®^.

## Future Directions for the Investigation of Acellular Extracellular Matrix Bioscaffold Therapy as an Adjunct to CABG

As described above, the mechanical environment in which an acellular ECM bioscaffold is employed plays a role in determining its optimal surgical indication. The ideal mechanical properties, such as stiffness or elasticity (Young’s modulus), of an acellular ECM bioscaffold for use as an adjunct to CABG remain unknown. For example, Peri-Guard^®^ (mean Young’s modulus, 95.67 MPa) exhibits greater stiffness or less elasticity compared to CorMatrix^®^, PhotoFix^TM^, and CardioCel^®^ (mean Young’s modulus, 36.78, 33.50, and 50.21 MPa, respectively) (Neethling et al., 2018). Further investigation is required to understand whether a more elastic acellular ECM bioscaffold, which may better approximate the native mechanical properties of the heart, is favorable to a less elastic acellular ECM bioscaffold that may provide greater structural support.

Additionally, the influence of acellular ECM bioscaffold therapy on the electrophysiology of the heart must also be considered. While the specific details are beyond the scope of this review, cardiac fibrosis is known to potentiate cardiac arrhythmia (Ten Tusscher and Panfilov, 2007; Pellman et al., 2016;Nguyen et al., 2017). By reducing cardiac fibrosis and improving microvascular perfusion of the infarcted myocardium, acellular ECM bioscaffold therapy may improve electrical conduction post-MI. In the case of CorMatrix^®^, our group has shown its bioinductive properties are responsible for stimulating endogenous mechanisms of vasculogenesis and functional recovery post-MI (Mewhort et al., 2014, 2016b, 2017). Further, FGF-2 enhanced-CorMatrix^®^ has been shown to improve contractility and exhibit positive electrical conductivity in a porcine model of MI sixty-days after surgical implantation (Tanaka et al., 2015). Clearly, leveraging acellular ECM bioscaffold therapy to improve electrical conductivity in the infarcted myocardium is an important component in determining its utility as an adjunct to CABG.

Beyond those discussed above, there are many other acellular ECM bioscaffolds used or in development for use not only in cardiac surgery, but across a variety of other surgical applications (Table 1). In addition to porcine and bovine sources, acellular ECM bioscaffolds may be also be sourced from equine or human donors (Table 1). Further, these bioscaffolds may specifically be derived from a variety of other tissue sources including the dermis, liver, and placenta (Table 1). Perhaps, as it is a highly regenerative organ, acellular ECM bioscaffolds derived from the liver may retain the native structural and physiological properties necessary to drive pro-reparative mechanisms *in-situ*. Alternatively, as the placenta is a highly vascularized structure, it may be that an acellular ECM bioscaffold derived from placental tissue holds the necessary bioinductive cues to promote neovascularization and integration of the bioscaffold into the native cardiac ECM microenvironment itself. Overall, as the composition and structure of ECM bioscaffolds are a function of the physiological requirements of the tissue from which they are derived, future work should consider the therapeutic potential of these alternative tissue-sources in developing acellular ECM bioscaffolds for use in cardiac surgery.

Ultimately, our research group is interested in investigating the use of acellular ECM bioscaffolds to influence and direct the local cardiac ECM microenvironment at the site of MI. The goal is to leverage this innovative technology to drive endogenous mechanisms of repair, such as angiogenesis or vasculogenesis, and to attenuate the activation of cardiac myofibroblasts. In doing so, the maladaptive structural cardiac remodeling that ensues following ischemic injury to the heart may be better managed. There is a gap in knowledge regarding the application of currently available acellular ECM bioscaffolds as an adjunct at the time of CABG surgery. Similarly, little is known about the ability of these bioscaffolds to influence the dysregulated ECM microenvironment of the damaged myocardium, possibly by way of retained bioactive properties and/or mechanical support. Therefore, there is an opportunity to characterize the potential therapeutic benefits of acellular ECM bioscaffold-based infarct repair.

We describe the varied clinical outcomes of currently available acellular ECM bioscaffolds in various cardiac surgery applications. We believe that initial investigations of the utility of acellular ECM bioscaffolds for infarct repair should begin with rigorous *in-vitro* testing. These studies should be complemented with well-designed pre-clinical investigations, which can be used to assess specific indications of ECM bioscaffolds. Finally, there is a need for larger scale prospective clinical trials to evaluate the possible therapeutic benefits of ECM bioscaffolds.

## Conclusion

A unique opportunity exists to leverage acellular ECM bioscaffolds for infarct repair. By implementing ECM bioscaffold therapy to modulate the local ECM microenvironment at the site of an MI, we could prevent maladaptive structural cardiac remodeling that often leads to irreversible heart failure. Future investigations should focus on the ability of these bioscaffolds to drive endogenous mechanisms of repair and to attenuate cardiac fibrosis. An ideal acellular ECM bioscaffold for infarct repair will be able to elicit functional recovery of the damaged myocardium. Acellular ECM bioscaffolds could have a significant clinical benefit and improve the prognosis of patients suffering from cardiac fibrosis and heart failure.

## Author Contributions

All authors designed, drafted, and revised the manuscript.

## Conflict of Interest Statement

The authors declare that the research was conducted in the absence of any commercial or financial relationships that could be construed as a potential conflict of interest.
